# Regulatory Hotspots in the Malaria Parasite Genome Dictate Transcriptional Variation

**DOI:** 10.1371/journal.pbio.0060238

**Published:** 2008-09-30

**Authors:** Joseph M Gonzales, Jigar J Patel, Napawan Ponmee, Lei Jiang, Asako Tan, Steven P Maher, Stefan Wuchty, Pradipsinh K Rathod, Michael T Ferdig

**Affiliations:** 1 The Eck Institute for Global Health, Department of Biological Sciences, University of Notre Dame, Notre Dame, Indiana, United States of America; 2 Department of Chemistry and Global Health, University of Washington, Seattle, Washington, United States of America; 3 Northwestern Institute of Complexity, Northwestern University, Evanston, Illinois, United States of America; Cornell University, United States of America

## Abstract

The determinants of transcriptional regulation in malaria parasites remain elusive. The presence of a well-characterized gene expression cascade shared by different Plasmodium falciparum strains could imply that transcriptional regulation and its natural variation do not contribute significantly to the evolution of parasite drug resistance. To clarify the role of transcriptional variation as a source of stain-specific diversity in the most deadly malaria species and to find genetic loci that dictate variations in gene expression, we examined genome-wide expression level polymorphisms (ELPs) in a genetic cross between phenotypically distinct parasite clones. Significant variation in gene expression is observed through direct co-hybridizations of RNA from different P. falciparum clones. Nearly 18% of genes were regulated by a significant expression quantitative trait locus. The genetic determinants of most of these ELPs resided in hotspots that are physically distant from their targets. The most prominent regulatory locus, influencing 269 transcripts, coincided with a Chromosome 5 amplification event carrying the drug resistance gene, *pfmdr1*, and 13 other genes. Drug selection pressure in the Dd2 parental clone lineage led not only to a copy number change in the *pfmdr1* gene but also to an increased copy number of putative neighboring regulatory factors that, in turn, broadly influence the transcriptional network. Previously unrecognized transcriptional variation, controlled by polymorphic regulatory genes and possibly master regulators within large copy number variants, contributes to sweeping phenotypic evolution in drug-resistant malaria parasites.

## Author Summary

Development of the malaria parasite, Plasmodium falciparum, in the blood is driven by a number of different genes expressed at different times and at different levels. Exactly what influences such transcriptional changes remains elusive, particularly in regard to important phenotypes like drug resistance. Using cDNA microarray hybridizations from the progeny of a *Plasmodium* genetic cross, we mapped gene expression quantitative trait loci (eQTLs) in an experimental population of malaria parasites. Each gene's transcript level was used as a segregating phenotype to identify regions of the *Plasmodium* genome dictating transcriptional variation. Several regulatory hotspots controlled the majority of gene expression variation, mostly via *trans-*acting mechanisms. One, influencing the largest number of transcripts, coincided with an amplified region of the genome traditionally associated with multiple drug resistance (MDR). Overall, integration of two functional genomic tools (gene mapping and transcript quantitation) has revealed a system-wide rewiring of the parasite transcription network: pleiotropic phenotypic variation, driven by drug selection on genome structure that may be attributed in large part to adaptive copy number polymorphisms in the parasite. These structural variants alter the expression of genes within the amplicon as well as many genes scattered across the *Plasmodium* genome.

## Abstract 3

Heritable levels of transcriptional variation, predominantly regulated by genomic copy number variants via trans mechanisms, are surprisingly abundant in drug-resistant malaria parasites.

## Introduction

Plasmodium falciparum is an apicomplexan parasite that causes the most severe and lethal form of human malaria. Parasites isolated from patients across the globe exhibit a wide range of phenotypic variation, including drug responses, growth rates, and a variety of virulence factors. Until recently, inter-strain variation had been studied primarily at the DNA sequence and phenotype levels. Since the P. falciparum genome was fully sequenced [1], several large-scale gene expression studies [2–8] have provided the malaria research community with detailed insights into gene expression across the parasite's life cycle. The P. falciparum transcriptome is expressed as an unusual continuous cascade across the distinct stages of the parasite's erythrocytic cycle [4]. However, the lack of traditional DNA-binding proteins raises important questions regarding the nature of transcriptional regulation, characteristically dictated by specific transcription factors in other eukaryotic systems [9,10]. While malaria parasites must have a complex regulatory architecture to control the precise waves of gene expression during erythrocytic development, it is not known whether natural allelic diversity in this species includes variations in the regulatory network itself. P. falciparum is not amenable to many of the standard tools employed to study model organisms. However, combinations of the few available genome-wide methods, including classical genetics, offer novel opportunities to dissect layers of regulatory complexities such as DNA copy number variation (CNV) and transcription.

Transcription in malaria parasites is rigidly programmed through the erythrocytic cycle and largely unresponsive to specific perturbation [11,12]. Recently, it was concluded that the parasite “lacks ubiquitous heritable transcriptional variation” [13], based primarily on recent work comparing gene expression profiles between three unrelated, lab-adapted parasite strains (i.e., parasite clones): 3D7, HB3, and Dd2 [6]. If true, it follows that divergent phenotypes between strains, such as drug resistance, do not result from variation in the transcriptional profile. This interpretation runs contrary to small datasets on direct co-hybridization of cDNA from malaria parasites [3], recent evidence that malaria parasites display distinct physiological states in their in vivo transcriptional profiles [14], and numerous observations that modifications in transcriptional regulation underpin complex adaptations in human, fly, worm, and yeast clonal populations [15–18].

Genetic mapping of genome-wide RNA levels as traits [19,20] has been applied to a variety of organisms to map regulators of transcription [16,21–27]. As has been observed for other classical phenotypes, transcript levels are inherited as complex traits that can be mapped to their causal genetic polymorphisms and, consequently, can broaden our understanding of the regulatory mechanisms underpinning adaptation to fluctuating environments.

Mapping the regulatory determinants of gene expression can be particularly useful in malaria parasites. The P. falciparum genome encodes much of the basal eukaryotic transcriptional machinery, including RNA polymerase II [1], and putative orthologs to general transcription factors have been identified [28]. However, for the most part, the functional roles of even these standard transcriptional regulatory components have not been experimentally confirmed. Comprehensive searches of Plasmodium spp. proteomes for specific transcription factors have yielded few candidates [29], with the exception of the identification of members of a candidate transcription factor ApiAP2 gene family [30]. A recent study by De Silva et al. (2008) [31] provides empirical support for ApiAP2 transcription factors and their cognate binding sequences as a potential source of developmental regulation in this species. The extremely AT-rich (> 80%) P. falciparum genome may obscure signatures of upstream or downstream regulatory motifs (e.g., [32]), as well as regulatory proteins, increasing the difficulty of identifying regulatory determinants in the genome. To compliment in silico searches for potential regulatory domains, an unbiased genetic approach can reveal components of the seemingly unique transcriptional regulation network in this important eukaryotic pathogen.

By mapping genome-wide variations in transcript levels, it is possible to describe a broad architecture of regulatory variation. Mapping transcript level traits to gene expression quantitative trait loci (eQTL) identifies “local” or “distant” genetic contributions for which a regulatory polymorphism resides near the target transcript's gene, or the regulatory variation is displaced from the gene's position, respectively [13]. On the genome scale, many different transcripts mapping to a single distant eQTL suggests a transcriptional regulator with multiple targets. Multiple expression traits that map to a common local eQTL point to either a local *cis* mechanism (e.g., polycistronic-like transcription) or a local structural determinant of gene expression (e.g., chromatin organization or a CNV such as a chromosomal amplification or deletion that alters the dosage of genes in a given locus).

Traditional quantitative trait loci (QTL) mapping in the HB3 × Dd2 genetic cross of malaria parasites identified the major candidate gene responsible for chloroquine resistance [33] and multiple QTL and candidate genes contributing to quinine susceptibility [34]. HB3, isolated from Central America (Honduras), represents a “wild-type” parasite sensitive to the quinoline line of drugs [35], while Dd2 was derived from a Laotian patient in whom chloroquine (CQ) therapy failed [36]. Dd2 is also resistant to pyrimethamine, as is typical of multidrug resistant (MDR) parasites. Moreover, prior to its use in the genetic cross, Dd2 was further selected in the laboratory for resistance to mefloquine (MQ) [37,38]; consequently the Dd2 genome has been reshaped by sequential drug selections and carries a genetic signature of, and effectively models, southeast Asian MDR parasites. The progeny from the HB3 × Dd2 genetic cross present a unique population in which to study the effects of drug selection on the parasite genome and its impact on gene expression. Understanding how a parasite's drug selection history has impacted its genetic plasticity has been the focus of persistent research [39] to devise sustainable drug therapies.

Here, we use genome-wide expression profiling and linkage analysis in the segregating population of P. falciparum derived from the Dd2 × HB3 genetic cross to locate regions of the genome contributing to heritable levels of transcriptional variation that distinguish parasite strains. We show that transcript level variation is strongly influenced by parasite genotype, and the controlling eQTLs are distributed throughout the genome. Regulatory loci are observed both proximal to and distant from the genes they regulate. They reveal the profound influence of structural chromosomal polymorphisms underlying the identified eQTLs. Overall, we have taken the first step toward understanding how groups of genes are co-regulated. By combining genomic methods with classical genetics, we illuminate previously unrecognized transcriptional complexity and variation in P. falciparum, including a prominent role for drug selection and CNVs in shaping the regulation of transcript levels and their downstream constituents.

## Results

### Genome-wide Expression Level Polymorphisms

**Figure 1:**
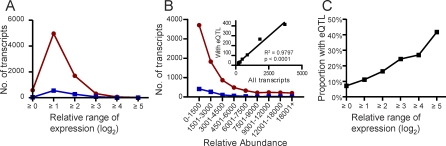
Genes with eQTLs as a Proportion of all Transcripts (A) The range of relative transcript levels between the highest and lowest expressing progeny clones (relative to the HB3 reference) observed for each transcript represented on the microarray at binned intervals (*x*-axis). Red circles represent all of the probes present on the array; blue squares represent the number of those that mapped as significant eQTLs (*p* ≤ 0.05). eQTLs were detected across the spectrum of relative transcript ranges in the progeny. (B) Relative transcript abundance at binned intervals (*x*-axis), plotted against probe counts for all probes represented on the microarray (red circles) and for all of those probes mapping to eQTLs at a genome-wide significance of *p* ≤ 0.05 (blue squares). The inset shows the strong linear relationship between the two groups of probes, demonstrating the consistent ability to identify eQTLs across the range of transcript abundances. (C) Percentage of probes that generated eQTLs at each defined relative range of expression levels in the progeny. An increase in proportion of probes with eQTLs was observed as the range of transcript's relative expression levels increased. More than 10% of the probes that exhibited a 2-fold or less range in relative expression levels among the progeny were mapped to at least one eQTL, highlighting the sensitivity of this method.

Using DNA microarrays, relative gene expression levels were measured across a genetic dimension in 34 progeny from a genetic cross of two laboratory adapted parasite strains. A high-resolution linkage map is available for this cross, and previous efforts have led to the mapping of genes involved in simple and multi-gene drug resistances [33,35,40]. For each progeny clone, relative transcript levels were determined for 7,665 probes representing 5,150 putative genes (open reading frames [ORFs]). Experimental precision derives from nested biological replication: each parental allele for each probe was represented on approximately 18 different microarrays given the predominant 1:1 mendelian segregation of markers genome-wide [40]. Malaria parasites are haploid during the erythrocytic stages and the impact of genotype on phenotype is more direct than for organisms in which influence from heterozygous loci must be taken into consideration. Extensive expression level polymorphisms (ELPs) appeared to be segregating in this population based on the range of relative transcript level variation observed for the vast majority of genes (Figure 1A).

### eQTL Mapping

Relative gene expression levels, reported as log_2_(^test^/_HB3 reference_), for all 34 progeny clones were used as expression traits for mapping eQTL. Transcript levels that vary due to nongenetic sources, such as biological or experimental noise, would not generate significant eQTL; however, performing the large number of tests required in eQTL studies increases the chance of obtaining a type I error. To account for the 329 microsatellite markers tested in the linkage analysis, 1,000 permutations were conducted for each eQTL scan (see Methods) to establish corrected genome-wide significance levels of *p* ≤ 0.05 and *p* ≤ 0.01 for each expression trait. All nominal and corrected *p*-values are provided in Table S1. A total of 874 ORFs (981 probes) was differentially regulated by 1,063 significant eQTLs at a genome-wide significance level of *p* ≤ 0.05 (Tables 1 and S1); approximately 18% of all P. falciparum genes displayed a significant genetic component leading to variation among the progeny at 18 h post-erythrocyte invasion (hpi). We also considered a genome-wide significance level of *p* ≤ 0.01 and found 315 expression traits with significant linkage (Table 1). In addition to correcting for multiple tests across the large number of markers for each expression trait, we computed false discovery rates (FDRs) associated with the genome-wide (corrected) *p*-value thresholds to account for the testing of 7,665 expression traits (Figure S1). The 981 (*p* ≤ 0.05) and 315 (*p* ≤ 0.01) expression traits regulated by eQTLs corresponded to FDRs of 24% and 14%, respectively. Although this study was performed in a non-model organism with relatively few progeny, the observed FDRs are consistent with observations from model genetic systems (e.g., [25,27]).

**Table 1:**
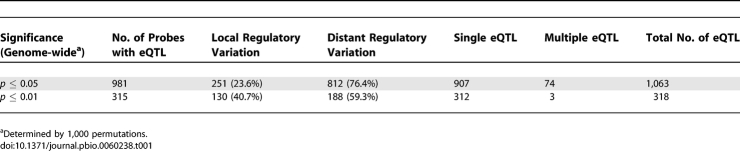
Numbers and Effects of Genome-wide eQTL

Nine hundred seven expression traits mapped to a single locus, and 74 traits mapped to multiple loci. Mapping in a small progeny population of 34 individuals might be expected to have limited power to detect multiple QTL for a given trait; however, this methodology was previously implemented for these same progeny to map five QTL contributing to quinine sensitivity and genetic effects accounting for as little as 10.5% of the phenotypic variation [34]. Nevertheless, the 74 traits for which multiple loci were identified could be disproportionately represented in the pool of expected false positives. In the present study, the detection of eQTLs was not obviously biased for particular phenotype characteristics. For example, eQTLs were detected across a range of relative expression levels among the progeny and span the range of transcript abundances (Figure 1A and 1B, blue squares). This illustrates the strength of the segregation filter and the high degree of nested replication among these progeny. Indeed, we detected loci for expression traits that varied by as little as 1.65-fold between the lowest and highest expressing progeny. Most eQTLs were detected for traits whose relative expression levels in the progeny varied 1 ≥ log_2_(^test^/_HB3 reference_) ≥ 2 (see Methods). As observed for the range in relative expression levels in the progeny, a large majority of eQTLs was derived from low abundant transcripts, and there was no skewing of eQTL detection toward the higher-abundance transcripts (Figure 1B, inset). This observation suggests that technical issues, such as labeling efficiency bias, were not influencing the global picture of eQTLs. As expected, traits with the widest range of variation among the progeny were more likely to have a significant eQTL (Figure 1C).

By considering eQTL numbers, positions, and effect sizes, it was possible to gain a sense of the potential regulatory complexity controlling expression traits. Local regulatory variation, wherein the causal locus of differential gene expression overlapped with the gene being regulated, accounted for 23.6% of the observed eQTLs (Table 1). The remaining majority (76.4%) of the eQTLs denoted mutations that regulated distant transcripts. A comprehensive list of genes and their eQTLs detected at different thresholds is provided in Table S2. Of the traits mapping to a single eQTL, 24% were local effects, commensurate with the overall eQTL pool. These were also the strongest genetic effects, as discerned by examination of the *p* ≤ 0.01 genome-wide eQTL threshold for detection (Table 1). Conversely, of the 74 probes that mapped to multiple loci, the most common (62% of the 74 probes) mapped to at least two distant eQTLs. It should be noted that such patterns of multi*-“trans”* factor regulation is much more difficult to detect in a small mapping population; therefore, while our data suggest complex *“trans”* regulators, we were limited to detecting only the largest genetic effects, no doubt underestimating the genetic complexity of transcriptional regulation in this species.

### Global Distributions of eQTLs

**Figure 2:**
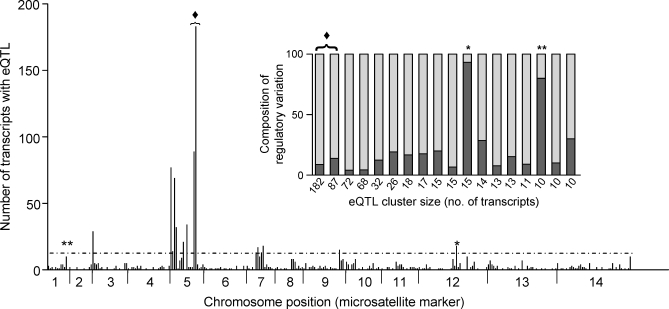
Genome-wide Distribution of eQTLs Counts of significant eQTLs are represented for each marker interval across the genome. The dashed horizontal line denotes the 95% confidence threshold for regulatory hotspots, calculated from 1,000 permutations (see Methods). Chr 5 accounted for nearly 50% of the total regulatory variation in the genome. The inset shows the composition of each cluster containing ten or more genes that mapped to the same locus, including those that surpassed the threshold for eQTL hotspots (≥14 genes). Most of these loci regulated the expression of distant genes, but two sets, denoted by one (*) and two (**) asterisks, were composed of mostly local eQTLs. These “local sets” coincide with chromosomal structural events (deletion of a locus at the end of Chr 2 [64] and an amplification event on Chr 12 [41]). The diamond (♦) denotes the two largest hotspots that aligned with the *pfmdr1*-containing amplicon of Dd2 on Chr 5 [41].

In addition to numbers and effect sizes, the genome-wide distribution of eQTLs can identify the regulatory architecture driving expression variation. Regulatory loci resided on each chromosome, ranging from as few as 17 eQTLs on Chr 6 to 513 on Chr 5 (Figure 2; Table 2). Of the 329 informative positions in the genome defined by recombination in the combined progeny pool [40], 203 loci harbored at least one eQTL, and 122 had multiple eQTLs (Figure 2). The 81 genome positions with a single eQTL (singletons) influenced expression of 32 local genes and 49 distant genes. Local effects were more common than distant effects in the singleton group (40% versus 24% of the total eQTLs, respectively). This could be because local eQTLs contributed to larger genetic effects and were thus more readily detected.

**Table 2:**
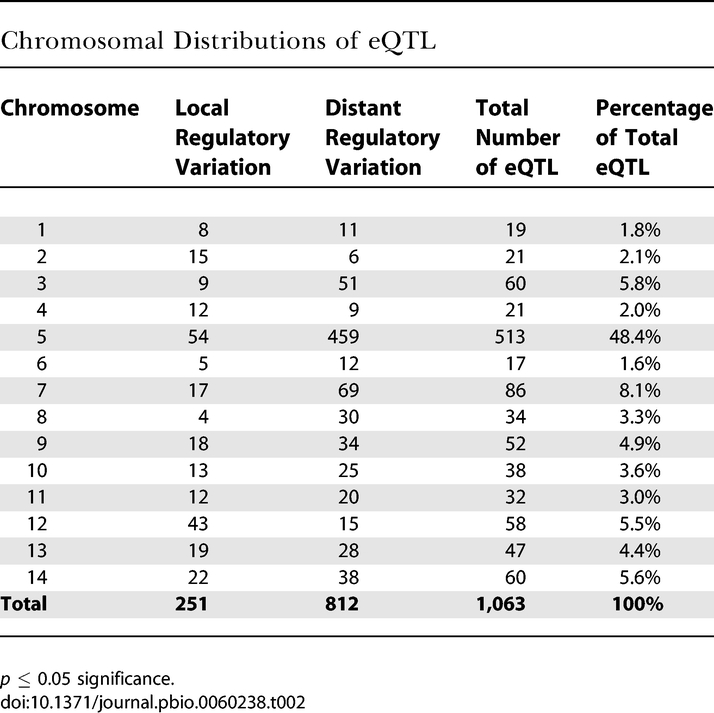
Chromosomal Distributions of eQTL

### eQTL Hotspots

Several loci influenced the expression of a very large number of genes across the P. falciparum genome. Using permutation tests (*p* ≤ 0.05; *n* = 1,000), we identified 12 regulatory hotspots (see Methods), each of which drove transcriptional changes of as few as 14 genes or as many as 182 genes (Figure 2; Table 3). These hotspots accounted for 63% of the detected eQTLs. More than half of the eQTL hotspots (seven of the 12) were found on Chr 5 (Table 3). These included the two largest eQTL hotspots that mapped to adjacent positions on Chr 5 (68.8 cM and 65.9 cM, respectively). The remaining eQTL hotspots were on Chr 3 (one hotspot), Chr 7 (two), Chr 9 (one), and Chr 12 (one). eQTL positions are defined with respect to their nearest independently mapped microsatellite marker; however, for any one expression trait, the resolution of a locus is a function of the genetic recombination resolution (i.e., cM distance). Although the high recombination rate (15 kb/cM) in P. falciparum [40] can facilitate physical mapping resolution to within tens of kilobases, it was not possible to know the exact degree to which neighboring loci overlap physically. Some overlap was expected, and this overlap influenced the designation of the total number of hotspots but did not influence the overall numbers of transcripts regulated by the adjacent segments. Consequently, under different criteria, neighboring loci would combine to generate fewer regulatory hotspots with more associated expression traits per hotspot. For example, when regulatory hotspots at adjacent markers were grouped together, the number of hotspots decreased to six, with hotspots 5_0.0 and 5_5.7 (Chr number_cM distance of genetic marker), 5_11.4 and 5_20.0, and 7_20.2 and 7_28.9 coalescing to three hotspots. Because this distribution of hotspots was critical to our interpretation of novel regulatory features of P. falciparum, we further compared the distribution of all eQTLs identified at the *p* ≤ 0.05 genome-wide significance threshold to those at identified at *p* ≤ 0.01and found a highly similar pattern of genome-wide eQTL clusters (*R* ^2^ = 0.9229) and retained seven of the 12 total eQTL hotspots (3_0.0, 5_0.0, 5_11.4, 5_20.0, 5_65.9, 5_68.8, and 12_103.3). Notably, some eQTL hotspots coincided with structural copy number variations that define their probable physical limits (described below).

**Table 3:**
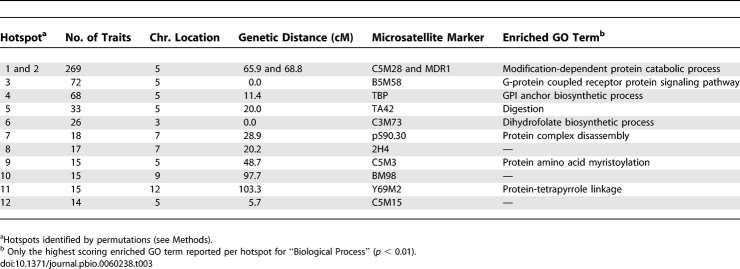
Regulatory Hotspots and Associated GO Term Enrichments

### *Trans-* Versus *cis-*Acting eQTL Hotspots

eQTL hotspots consisting of distant regulatory effects point to pleiotropic regulators [16]. Alternatively, eQTL hotspots composed of many local eQTLs likely result from chromosomal structural events, such as sequence amplifications or deletions that impact the expression of resident genes. *Cis*- and *trans*-acting hotspots may coincide if, for example, a deletion includes transcription factors that act at distant sites, i.e., the genes within the deletion will have reduced transcription as will the distant genes they regulate. The vast majority (86%) of the “linked” eQTLs in P. falciparum hotspots regulated the transcription of unlinked genes. Only a single hotspot (Chr 12) was found to correspond to mostly local regulation of transcripts. Furthermore, when considering a reduced eQTL hotspot threshold (ten or more co-mapping expression traits), we uncovered one additional locus with a high proportion of local genes (Chr 2), compared to an additional five loci corresponding to distant gene regulation (Figure 2, inset).

### Gene Amplifications, Deletions, and eQTL Hotspots

It was of interest to compare the eQTL hotspots with previous comparative genome hybridization studies that identified chromosomal amplifications and deletions in the parental P. falciparum clones. The two eQTL clusters in the present study that predominantly regulated local gene expression (Figure 2, inset, see asterisks) included a Chr 12 hotspot coinciding with a CNV in the underlying DNA segments [41]. Three hotspots aligned with sequence amplification events (eQTL hotspots 5_65.9, 5_68.8, and 12_103.3) [41–43] and one hotspot (9_97.7) corresponded to a deleted segment from the HB3 parent [44]. Four hotspots were linked to loci rich with cytoadherence and highly polymorphic surface antigen genes such as *cytoadherence linked asexual protein* (*CLAG*) genes, *rifin* genes, and *var* genes (3_0.0, 5_0.0, 5_5.7, and 7_28.9, respectively) [1], of which three were located in the sub-telomeres and one (7_28.9) at an internal (chromosomal) *var* cluster [45,46]. One hotspot (7_20.2) was observed near the *pfcrt* drug-resistance locus, and the remaining three (5_11.4, 5_20.0, and 5_48.7) did not coincide with a previously identified CNV, drug resistance locus, or other highly polymorphic region of the genome.

###  Allelic Effects Associated with the Chr 5 Amplification Event

**Figure 3:**
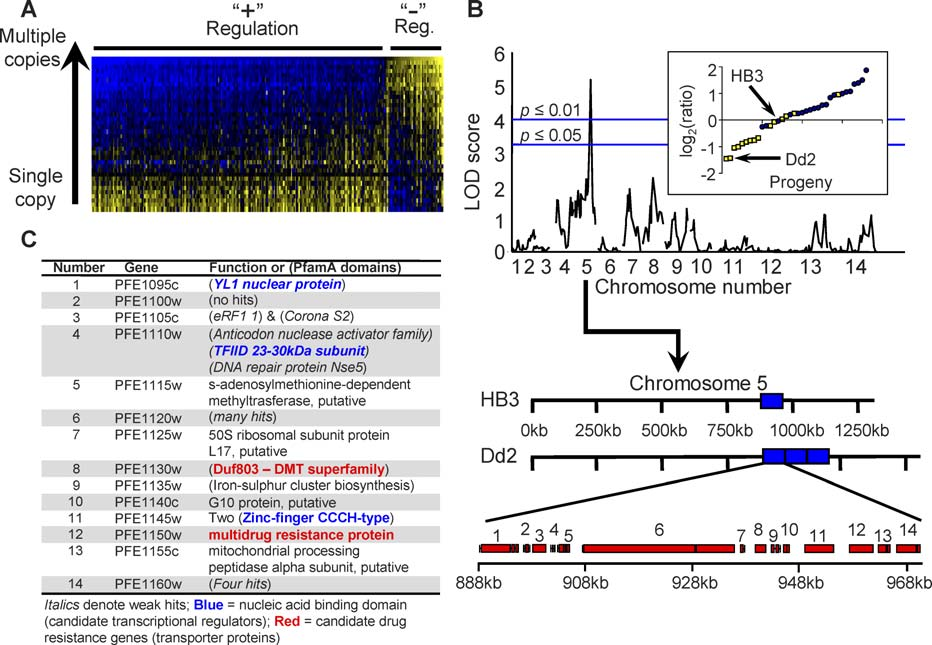
Transcript Levels Regulated by the *pfmdr1* Amplicon on Chromosome 5 (A) Heatmap of relative gene expression levels for 269 transcripts that mapped to the two markers corresponding to the Chr 5 amplicon containing *pfmdr1* (see Figure 2, diamonds). Transcripts are aligned along the *x*-axis and progeny are aligned on the *y*-axis. The transcripts have been ranked from highest to lowest relative expression value for the Dd2 parent (top row). Progeny are ranked by their correlation to Dd2 (i.e., the bottom row is the least correlated to Dd2). Progeny that inherited a single copy of the amplicon (i.e., the HB3 parental allele) at this regulatory locus exhibited a similar expression pattern to the HB3 parent. Progeny that inherited multiple copies of the amplicon had relative expression values more similar to Dd2. Nearly all of these transcripts are distant from the amplicon (Figure 2, inset). Approximately 85% of the transcripts were positively regulated (+); inheritance of multiple copies of the regulator corresponded to increased transcription (blue). Conversely, approximately 15% of the transcripts were negatively regulated (−); inheriting multiple copies of the regulator decreased transcript levels (yellow). (B) As an example, the eQTL mainscan for PFI0290c, a gene residing on Chr 9 whose regulation mapped to the Chr 5 amplicon. The inset illustrates the relative expression of this gene in the progeny clones; progeny inheriting the Dd2 parental allele are shown as yellow squares and those inheriting the HB3 parental allele shown as blue circles. The arrow from Chr 5 on the mainscan axis points to a schematic view of the Chr 5 amplicon and resident genes. In the Dd2, the selection for multiple copies of *pfmdr1* has led to the selection of multiple copies of 13 other genes linked to *pfmdr1*. (C) Nine of the 14 genes are characterized as having “unknown function” in the database. Consequently, we conducted domain searches using the PfamA database to identify possible transcriptional regulators. One gene, PFE1145w, containing two tandem zinc finger domains, characteristic of a nucleic acid binding domain common to transcription factors, was identified as a primary candidate transcriptional regulator.

To assess the genetic basis for transcriptional variation mapping to the Chr 5 amplicon (markers 5_65.9 and 5_68.8), we evaluated the relative gene expression levels within the progeny of the HB3 × Dd2 cross (Figure 3A). These loci span the previously reported amplification event involving the multiple drug resistance gene, *pfmdr1* (PFE1150w), found in Dd2 but not in HB3 [35,47]. We merged the genes whose transcripts map to these prominent eQTLs into a single set of genes for further analysis, because the posterior probability for the probes mapping to each of these markers generally spanned both markers and the entire amplicon.

Of the 269 transcripts mapping to the amplicon, 85% (228) were expressed at higher levels in the Dd2 parent compared to the HB3 parent; furthermore, these genes were expressed at higher levels in the progeny that inherited the Dd2 alleles at these loci, as expected for an overexpressed positive transcriptional regulator located within the amplicon. The remaining 15% (41) of the transcripts regulated by these hotspots were generally expressed higher in those individual genotypes inheriting the HB3 alleles at these loci. In the simplest scenario, this would argue for a *trans*-acting, negative regulator located in the amplified region. Clear segregation of the two allelic effects was evident among these transcripts (Figure 3A), demonstrating strong regulation associated with the number of copies of this amplicon carried by individual progeny. We were surprised to observe clear subsets of up-regulated and down-regulated genes due to this amplification. While there is no need to presume that the underlying mechanisms regulating these genes would necessarily lead to solely up-regulation, we considered the possibility that the down-regulated genes were more likely false positives. We observed no statistical difference between the *p*-values associated with genes regulated in each direction, and also found the same proportion of genes up- and down-regulated at the highly significant *p* ≤ 0.01 genome-wide eQTLs (unpublished data; see Table S1), supporting the validity of these two regulatory directions of transcription.

### Identifying Candidate Regulatory Factors

In Dd2, the *pfmdr1* amplicon contains 14 ORFs [41] and is repeated three times [47] (Figure 3B). This eQTL hotspot indicates that a polymorphism(s) in or linked to the amplicon is regulating genes across the genome; therefore, we closely examined genes in the amplicon for possible transcriptional regulators (Figure 3C). Of the 14 genes, nine are of unknown function, i.e., hypothetical proteins. We used the predicted amino acid sequences for each of the nine hypothetical proteins to search the PfamA database [48] for protein domains characteristic of DNA binding function, a domain function common to transcription factors. Three genes with significant hits to known protein domains were identified: PFE1130w had a hit to a Duf803 domain common to the drug metabolite transporter superfamily of genes (E-value = 0.0001), PFE1135w had a hit to a domain common to iron-sulfur cluster biosynthesis genes (E-value = 2.9 × 10^−24^), and PFE1145w had hits to two tandem zinc-finger CCCH-type domains (E-value = 0.18 and 0.00084), spaced ten amino acids apart. Two additional genes with unknown function (PFE1095c and PFE1110w) were identified with weaker hits to nucleic acid binding domains (YL1 nuclear protein and transcription initiation factor TFIID 23–30 kDa subunit, respectively). PFE1110w previously has been hypothesized to be a P. falciparum ortholog for TFIID TAF10, a basal transcription factor associated with RNA polymerase II activity in other eukaryotic systems [28]. These three genes, whose proteins encode putative DNA binding domains (PFE1110w, PFE1110w, and PFE1145w), are primary candidate genes with a role in transcriptional regulation requiring experimental validation.

### Co-regulated Genes with Shared Function

Distant regulatory variation accounted for most of the transcripts mapping to the eQTL hotspots, suggesting possible master regulators residing within the hotspots. To ascertain co-regulation of possible functionally related genes, we sought Gene Ontology (GO) enrichment (Table 3). Focusing on the Chr 5 *pfmdr1* amplification hotspot (5_65.9 and 5_68.8), we found that protein modification via the proteasome complex was a prominent, differentially regulated GO category within our experimental population (Table 4). Notably, four additional hotspots contained differentially regulated genes involved in protein modification (eQTL hotspots at 5_48.7, 7_28.9, 9_97.7 and 12_103.3), implying that transcriptional regulators of post-transcriptional machinery may play a prominent role in phenotypic differences between HB3 and Dd2 (Tables 3 and S2). This strong presence of genes involved in protein modification highlights a biological pathway that is alternatively regulated in the parental parasites' genetic backgrounds. Complete results for calculated GO enrichment values for each of the eQTL hotspots are provided (Table S3).

**Table 4:**
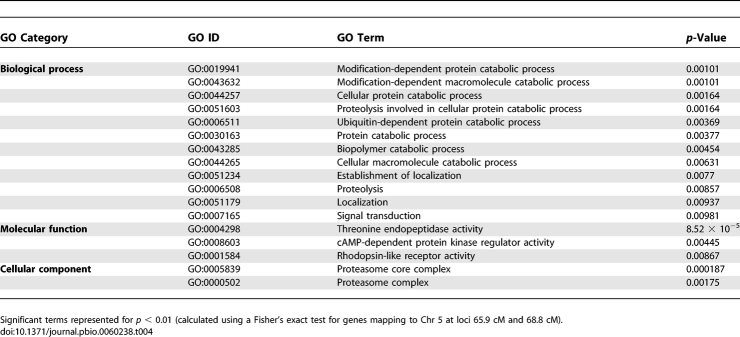
Functional Enrichments Associated with Chr 5 *pfmdr1* Amplification Hotspot

###  Cell Cycle Gene Expression Profiles

**Figure 4:**
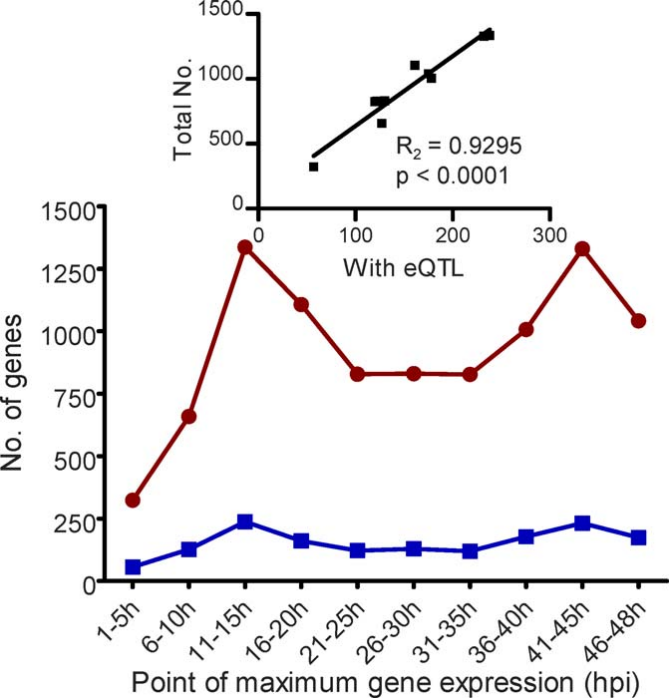
Genes with eQTLs as Represented by Peak Expression across the Parasite Life Cycle Genes whose expression peaks at defined hour intervals across the parasite life cycle (red circles) and the number of those genes that had a significant eQTLs (blue squares). The inset depicts the relationship between the two sets of gene counts, demonstrating that eQTLs were consistently found for genes whose expressions peak across the parasite life cycle and were not biased toward 18 hpi, the time at which samples were collected for this study.

HB3 and Dd2 are known to diverge in the duration of their erythrocytic cell cycle [6,49]; consequently, we evaluated the possibility that eQTLs corresponded to events of the cell cycle. We first binned genes represented on the microarray by their peak expression time in the life cycle using the same microarray platform [4,6]. We then compared these peak-time bins with genes regulated by eQTLs (Figure 4). No bias toward stage-specific gene expression was present in the eQTL pool.

## Discussion

An emerging theme in evolutionary biology recognizes that mutations in regulatory sequences can account for major physiological differences between strains even when coding genes are relatively unchanged [18]. While in numerous species variation in gene expression serves as a storehouse for phenotypic variation [16,21–23,50], it has been argued that P. falciparum is unusual in exhibiting remarkably little heritable, strain-to-strain variation [13]. This conclusion was based on a study by Llinas et al. that compared broad expression “cascades” across the complete erythrocytic cell cycle for three different parasite clones [6], including HB3 and Dd2. Their approach assessed transcriptional profiles by measuring a parasite's transcript levels against pooled samples from the same parasite clone and was not designed to directly assess the relative abundance of transcripts between strains. In contrast, the current study measured relative transcript abundance of the Dd2 parent and each progeny clone against a common reference, HB3, the other parent from the genetic cross. The two studies are not necessarily contradictory, but rather illuminate different features of transcription in this lethal malaria parasite species emphasizing both the robust transcriptional program that has been so well characterized in this species and the subtle but abundant variation that exists between strains. Viewing transcription across a genetic rather than a developmental dimension allows us to tease out variations in transcriptional regulation that could have important implications for the *Plasmodium* regulatory network and its role in adaptive evolution.

Until now, no study of malaria parasites (to our knowledge) has specifically examined the genetic inheritance of transcriptional variation. More generally, our work illustrates the concept that genome-wide readouts of segregating natural variation point to polymorphic regulatory loci, a finding particularly relevant in light of recent observations by Daily et al. (2007) [14] that transcriptional profiles associated with distinct metabolic states in blood stage forms of malaria are observed in parasites isolated directly from patient blood; these metabolic states are proposed to influence the course of infection and virulence. In the present study involving cultured parasites under controlled laboratory conditions, we find heritable variation in expression levels to be as extensive as that reported for other organisms. We also find transcription levels to be regulated by few, predominantly distant, eQTL hotspots. These co-regulated genes underpin altered biological processes of the regulatory network and provide an evolutionary path to phenotypic change that is distinct from potentially deleterious coding mutations that alter protein function and would thus be poorly tolerated in haploid parasites. Given the relatively small number of progeny available from the HB3 × Dd2 cross, the eQTLs presented probably capture only the largest genetic effects and certainly underestimate the total number and complexity of regulatory polymorphisms.

We find that a large proportion (76.4%) of the genes with variable transcript levels is influenced by distant regulators, dominated by several regulatory hotspots. Distant regulatory variation is often associated with *trans*-acting transcription factors. While P. falciparum has been described as having a paucity of transcription factors [1], De Silva et al. (2008) recently experimentally validated a family of specific transcription factors and their DNA binding sites [31], suggesting that the machinery for complex regulation in P. falciparum is present yet difficult to discern by standard homology searches. Our data support the presence of at least a few additional potent regulatory factors, and eQTLs can be dissected to locate candidate transcriptional regulating genes in apicomplexans [30]. Regulatory loci can be identified through this method irrespective of the specific biochemical functions (e.g., DNA binding, nuclear localization, or protein phosphorylation) and is therefore uniquely suited to identify loci harboring genetic determinants that act as traditional and atypical or unique modes of genome regulation.

Although most eQTLs identified in this study are *trans*-acting, the strongest genetic effects are due to local regulatory polymorphisms (Table 1). These polymorphisms are more likely to occur individually than in clusters. Local linkages arise from classical *cis*-acting mechanisms, e.g., a polymorphism in the gene regulatory region; various other scenarios are also possible, including CNVs, splicing, mRNA decay, regional chromatin structure [23], or even unconventional autologous protein–nucleic acid interactions [51]. Such *cis* elements would be expected to have strong genetic effects due to the direct molecular control of the transcript levels. We are aware that eQTL mapping has the potential to overestimate local regulatory variation if substantial sequence variation is present between alleles due to reduced hybridization efficiency, effectively mimicking lower gene expression levels [52]. However, for the divergence between HB3 and Dd2, less than 10% of the probes (expression traits) with eQTLs arose from the highly polymorphic antigenic gene families including *vars*, *rifins*, and *stevors*, nearly the same proportion of these genes represented in the overall set of 7,665 probes (10% versus 8%, respectively). In fact, we detected a relative paucity of local eQTLs compared to a major role for *trans*-acting mechanisms controlling the majority of the observed ELPs in the HB3 × Dd2 genetic cross. We find that genetic effects due to distant regulatory variation are smaller and more likely partnered, indicative of genetic complexity of the regulatory network. Regulatory hotspots have been hypothesized to contain “master regulators” with the effective mutations having pleiotropic effects [24,26], wherein one DNA sequence variant impacts multiple expression traits with potential broad impact on downstream classical phenotypes.

Another striking feature of the regulatory architecture is the prominent role of CNVs in directing transcription variation, particularly from regions previously associated with drug resistance traits. Originally, the HB3 × Dd2 cross was generated to study chloroquine resistance in malaria parasites [35]; fortuitously, the differing geographic and drug selection histories of these genomes represent independent solutions for survival in their respective environments. It is also likely that adaptations to intensive, long-term drug selection by CQ, and subsequent selections by pyrimethamine and MQ, broadly affect biological processes, perhaps pointing to physiological compensation of mutations in drug resistance genes. Our analysis demonstrates GO term enrichment for genes regulated by each of the eQTL hotspots. Genes related to post-transcriptional protein modification are, intriguingly, enriched in six of the 12 eQTL hotspots, including the two hotspots coinciding with the *pfmdr1* amplicon on Chr 5 (Tables 3 and 4), suggesting that physiological differences between the parental parasite clones, HB3 and Dd2, may be at least partially due to post-translational modification/regulation of proteins. This is interesting given the recent findings in yeast that protein levels were unchanged in individuals with increased aneuploidy despite increased gene expression levels, suggesting active regulation at the protein level [53]. These functional differences are prime candidate processes driving phenotypic differences derived from the genetic adaptations associated with multiple drug resistance in the Dd2 parent line and may shed light on post-transcriptional regulation of the genome key to adaptation in the malaria parasite.

DNA sequence amplification events, occurring as multiple tandem copies, can influence gene expression both locally and distantly through dosage effects. Previous laboratory drug selection studies demonstrated that parasites exhibited amplification of the genomic region carrying *pfmdr1* on Chr 5 that coincided with a loss of sensitivity to quinine and MQ [54]. Additionally, work performed on parasite isolates from MDR populations confirmed amplification of the *pfmdr1* locus is the primary contributor for resistance to MQ [55]. The focus of previous studies on this locus has been the *pfmdr1* gene itself, and the roles of coding mutations and gene dosage in influencing drug sensitivities [54–56]. Our data emphasize a potentially critical role of neighboring genes, e.g., increased copy numbers of the entire *pfmdr1*-containing locus not only coincides with decreased sensitivities to common antimalarial compounds but significantly alters gene expression throughout the genome. In addition, genes regulated by the Chr 5 amplicon are both positively and negatively regulated, potentially indicating multiple regulators or multiple mechanisms for gene regulation contained in this locus. Figure 3C illustrates transcriptional regulator candidates, PFE1095c, PFE1110w, and PFE1145w, residing on the *pfmdr1* amplicon, including a tandem zinc-finger domain protein (PFE1145w). Notably, eQTL hotspots are not confined to transcription factors but could also point to novel regulatory mechanisms.

The degree to which drug selection on malaria parasites can impact the expression of genes across the genome is evidenced by the sweeping affects the Chr 5 *pfmdr1*-containing amplicon has on genome-wide gene expression. In general, Chr 5 has been a primary target for genomic adaptations in the HB3 and Dd2 parental parasites; determining whether Chr 5 is unique to this parasite population or whether it is a key player in all natural parasite adaptations is relevant to understanding the propensity of certain parasite clones to rapidly become resistant to multiple drugs. Identifying the mechanisms of transcriptional regulation and the functional relationships among the co-regulated genes will provide crucial information regarding potential antimalarial targets. It is likely that the drug-selection history of Dd2 affecting relatively few “resistance” genes has had a broader impact with significant implications for parasite biology in the form of drug sensitivity modulators, compensatory mechanism in physiology, and/or simple hitchhiking effects that could themselves acquire an adaptive role in subsequent selection.

In light of historical selection bottlenecks and its impact on multi-drug resistance and virulence, even “simple” resistance mechanisms, e.g., the point mutation in the chloroquine resistance transporter gene *pfcrt*, conferring resistance to CQ, may elicit a complex expression signature. Here, we illustrated how eQTL scans can uncover nontraditional patterns of transcriptional regulation underlying strain-level variation and regulatory hotspots in P. falciparum. This study bolsters a significant and unexpected role for divergent transcription as a source of phenotypic variation and evolution in malaria parasites and elevates a role for structural changes (e.g., CNVs) as having potentially prominent consequences in the cell, perhaps contributing to adaptive evolution.

## Materials and Methods

### Genetic cross and parasite culturing.

Parent and progeny parasites (i.e., clones) of the HB3 × Dd2 genetic cross were obtained from the original cloned stocks [35]. The HB3 × Dd2 genetic cross consists of 35 haploid progeny (34 of which were available for this study), mimicking, in effect, recombinant inbred lines for linkage analysis. Each progeny was previously genotyped for the 329 informative microsatellite markers spanning the 14 chromosomes (23 Mb) at a resolution of approximately 17 kb/cM [40]. Parasites were cultivated in human erythrocytes (RBCs) by standard methods [57,58] utilizing leukocyte-free human RBCs (Indiana Regional Blood Center, Indianapolis, Indiana) suspended in complete medium (CM) [RPMI 1640 with L-glutamine (Invitrogen, Carlsbad, California), 50 mg/l hypoxanthine (Sigma-Aldrich, St. Louis, Missouri), and 25 mM HEPES (Calbiochem, San Diego, California); 0.5% Albumax II (Invitrogen), 10 mg/l gentamicin (Invitrogen), and 0.225% NaHCO_3_ (Invitrogen)] at 5% hematocrit. Cultures were maintained independently in sealed flasks at 37 °C under an atmosphere of 5% CO_2_, 5% O_2_, and 90% N_2_. Parasitemia was monitored and maintained at 5%–7%. Parasites were synchronized using two consecutive 5% sorbitol treatments for two generations, followed by one cell cycle without treatment. The 18 hpi time point was determined by light microscopy. Samples were flash frozen in liquid nitrogen prior to extraction of total RNA.

### Isolation of RNA, cDNA synthesis, and microarray hybridization.

Total RNA was isolated as previously described using TRIzol reagent [4]. cDNA was synthesized using the Ovation Aminoallyl RNA Amplification and Labeling Kit (cat. # 2101–12; NuGEN Technologies, San Carlos, California) and prepared for hybridization to microarrays as previously described [4]. Microarrays contained 7,665 70mer oligonucleotide probes representing 5,150 Plasmodium falciparum ORFs kindly provided by Dr. Joseph DeRisi (University of California, San Francisco, San Francisco, California). The majority of the oligonucleotides for printing the array corresponded to the Qiagen Operon set (Operon Biotechnologies, Huntsville, Alabama), but some additional sequences matched ORFs in the original P. falciparum sequence reads. The oligonucleotides were printed on polylysine-coated slides using a new generation, ultra fast, linear servo driven DeRisi microarrayer. Slides were post-processed and hybridized in 3× SSC at 63 °C for 12 h as previously described [4]. Slides were scanned in an Axon GenePix 4000B microarray scanner (Axon Instruments, Union City, California) with 532 nm (17 mW) and 635 nm (10 mW) lasers. Data were collected as an image file, gridded, and converted into a text file using Genepix 3.0 software (Axon Instruments). Each parental and progeny cDNA (labeled with Cy5) were hybridized to a common reference sample of the HB3 parental clone (labeled with Cy3), allowing for direct comparisons between the results from each microarray; we tested 34 progeny clones, for a total of 36 hybridizations.

### Data preparation, visualization.

Lowess smoothing of the raw expression data was used to normalize across all microarray slides using TIGR's MultiExperiment Viewer (MeV) v4.0 and Microarray Data Analysis System (MIDAS) v2.19 software (http://www.tm4.org/). Gene expression values are reported as log_2_(^sample^/_HB3 reference_) of each normalized fluorescent signal. The DecisionSite software (Spotfire, Somerville, Massachusetts) was used to generate relative gene expression heat maps.

### Linkage analysis.

For each expression trait, interval mapping linkage analysis was performed using the Bayesian approach implemented in the Pseudomarker v2.03 package [59] executed in MATLAB (The MathWorks, Natick, Massachusetts). Marker genotypes were used directly (i.e., imputation of “pseudomarkers” was not necessary given the high-resolution, uniform marker coverage of the P. falciparum genome [40]), and the marker corresponding to the highest LOD score for each expression trait was retained as the eQTL position. The empirical genome-wide significance was determined for each trait by permutations [60,61] in which progeny haplotypes were randomly associated with expression trait values and linkage analysis performed 1,000 times. The nominal *p*-values for each trait were converted to genome-wide corrected *p*-values using the permutation distribution of the maximum LOD score for each trait. This approach corrects the nominal *p*-values for the multiple testing of 329 markers. LOD score thresholds corresponding to *p* ≤ 0.05 and *p* ≤ 0.01 genome-wide significance thresholds were determined for each expression trait. To account for the multiple testing of 7,665 expression traits, we followed the work of Petretto et al. (2006) [27] in which we use the lowest corrected *p*-value for each expression trait to calculate the FDR, using the *q* value method of Storey and Tibshirani (2003) [62]. This approach was motivated by the fact that the underlying procedure to estimate the FDR works well under “weak” dependence of the considered features. To be conservative, an eQTL was classified as “local” if the gene was located on the same chromosome as the linkage or as due to “distant” regulatory variation if located elsewhere in the genome.

### Linkage hotspots and GO enrichment.

To locate regulatory hotspots within the genome, the total number of expression trait mapping to eQTL at the *p* ≤ 0.05 genome-wide significance threshold, specifically 1,063, were randomly assigned to the markers (loci) in the genome. The number of linkages observed at the locus with the most linkages was retained and this process was repeated 1,000 times creating a random distribution. From this distribution, linkage hotspots were identified as the number of traits mapping to a given locus exceeding the 95% distribution frequency derived from the 1,000 permutations (i.e., at *p* ≤ 0.05 for *n* = 1,000). Genetic markers harboring 14 or more traits with an eQTL were not expected by chance. Genes comprising the groups of genes at the eQTL hotspots were sifted for enriched GO terms to identify possible co-regulated genes contributing to the same biological processes. To obtain statistically significant measurements of overrepresented GO terms, we applied a Fisher's exact test as implemented in GOStat [63]. GO terms significant at *p* < 0.01 for each regulatory hotspot were retained and reported.

### Proportion of genes with eQTLs across the parasite life cycle.

The peak hours of gene expression for all genes represented on our microarrays were collected from prior microarray experiments using the same platform (DeRisi Lab Malaria Transcriptome Database; http://malaria.ucsf.edu/). Genes were binned at 5 h intervals based on their respective peak hour of expression across the HB3 and Dd2 parasites' life cycles. The percentage of genes whose probes showed significant linkage within each of the 5 h bins was calculated. Regression analyses were performed using GraphPad Prism v4.0 (GraphPad Software, La Jolla, California).

### Proportion of transcripts with eQTLs across various relative abundance levels.

Relative transcript abundance was qualitatively estimated for each probe on our microarray by using the sum of median (SOM) intensity from each array, similar to a previous study using the same microarray platform [6]. For each probe, the highest 18 SOM intensity values across all progeny were retained and the median was calculated. Each probe was then binned according to its corresponding relative transcript abundance. The percentage of probes showing significant linkage within each of the relative abundance bins was then calculated.

### Proportion of transcripts with eQTLs across different ranges of relative expression values.

The range of relative gene expression levels for each transcript was calculated from the progeny with the highest and lowest relative expression levels compared to the reference HB3 samples, excluding outliers. Outliers were determined as those progeny clones expressing at levels ± 2 standard deviations from the mean, calculated for each probe independently. Probes were then binned according to their relative expression level range in the progeny. The percentage of probes with significant linkage within each of the expression range bins was then calculated.

## Supporting Information

Figure S1 Relationship between Corrected eQTL *p*-values and Corresponding FDR A scatter plot representing the relationship between genome-wide eQTL *p*-values (*x*-axis) and the corresponding FDR (*y*-axis) as calculated using q values [62] to account for the multiple testing of 7,665 expression traits. The inset zooms in on the lower end of the *p*-value spectrum from the main graph.
(134 KB PDF)

Table S1 Nominal and Genome-wide Corrected *p*-Values for Each Expression Trait (258 KB CSV)

Table S2 eQTL Location Summary for the 7,665 Mapped Expression Traits at Each Detection Threshold (478 KB CSV)

Table S3 GO Term Enrichment for Genes Mapping to Regulatory Hotspots (37 KB XLS)

## Abbreviations

Chr: Chromosome
CNV: copy number variant
CQ: chloroquine
ELP: expression level polymorphism
eQTL(s): expression quantitative trait locus(i)
FDR: false discovery rate
GO: Gene Ontology
hpi: hour(s) post-invasion
MDR: multiple drug resistant
MQ: mefloquine
ORF: open reading frame
