# Supplementary figures and images for: Regulatory Hotspots in the Malaria Parasite Genome Dictate Transcriptional Variation
Source: PLoS Biol. 2008 Sep 30;6(9):e238. doi: 10.1371/journal.pbio.0060238 (PMC2553844; doi:10.1371/journal.pbio.0060238)

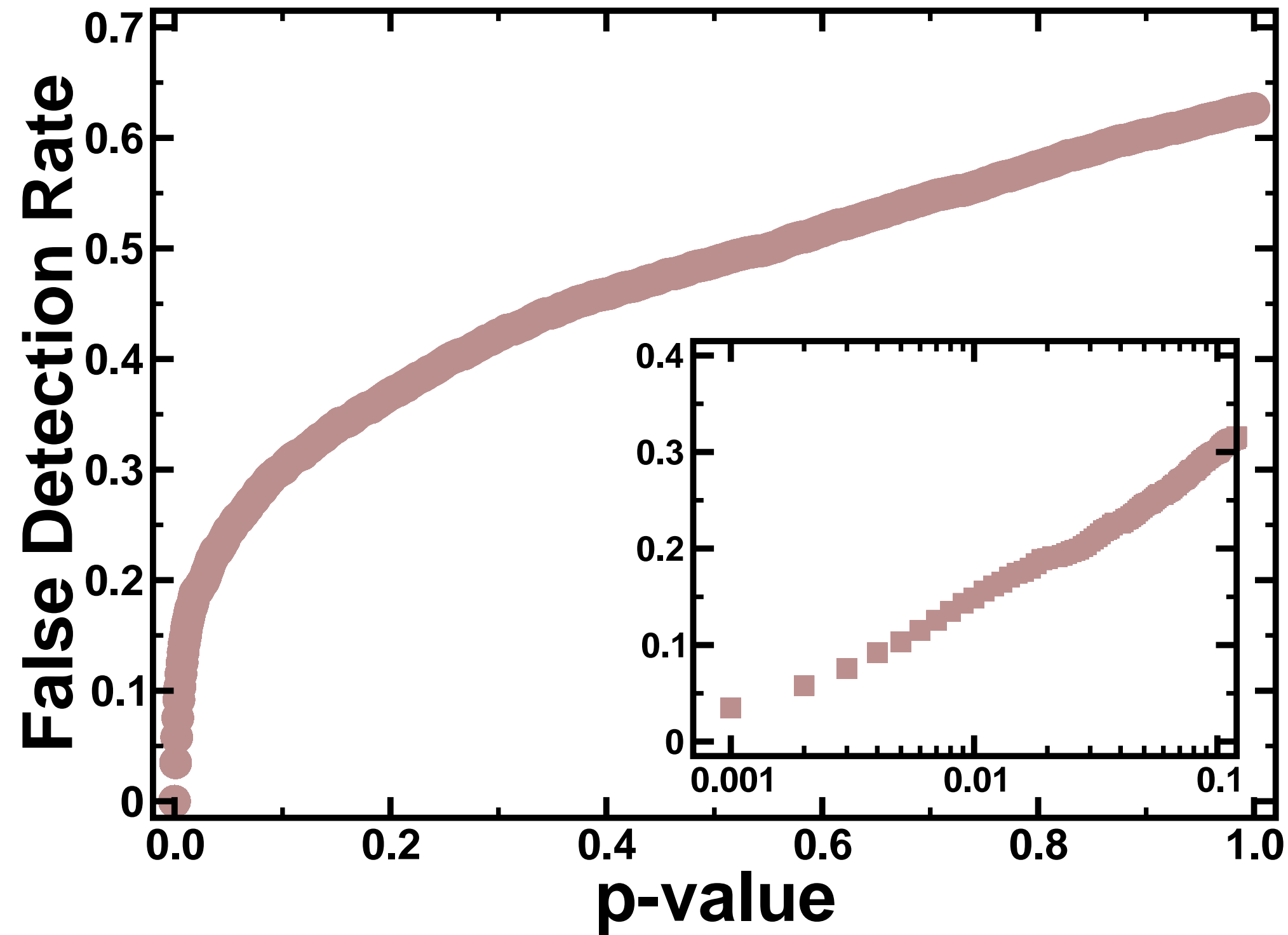

Supplement: Figure S1 — A scatter plot representing the relationship between genome-wide eQTL p-values (x-axis) and the corresponding FDR (y-axis) as calculated using q values [62] to account for the multiple testing of 7,665 expression traits. The inset zooms in on the lower end of the p-value spectrum from the main graph. (134 KB PDF) [file pbio.0060238.sg001.pdf]
